# Hereditary Neuropathy with Liability to Pressure Palsies Masked by Previous Gunshots and Tuberculosis

**DOI:** 10.1155/2015/738469

**Published:** 2015-11-11

**Authors:** Martin Gencik, Josef Finsterer

**Affiliations:** ^1^Human Genetic Laboratory, 1180 Vienna, Austria; ^2^Krankenanstalt Rudolfstiftung, Vienna, Austria

## Abstract

*Objectives.* Although hereditary neuropathy with liability to pressure palsies (HNPP) presents with a distinct phenotype on history, clinical exam, and nerve conduction studies, it may be masked if diagnostic work-up suggests other causes.* Case Report.* In a 37-year-old male with pseudoradicular lumbar pain, neurological exam revealed sore neck muscles, peripheral facial nerve palsy, right anacusis and left hypoacusis, hemihypesthesia of the right face, mild distal quadriparesis, diffuse wasting, and generally reduced tendon reflexes. He had a history of skull fracture due to a gunshot behind the right ear and tuberculosis for which he had received adequate treatment for 3 years; MRI revealed a disc prolapse at C6/7 and Th11/12. Nerve conduction studies were indicative of demyelinating polyneuropathy with conduction blocks. Despite elevated antinuclear antibodies and elevated CSF-protein, HNPP was diagnosed genetically after having excluded vasculitis, CIDP, radiculopathy, and the side effects of antituberculous treatment.* Conclusions.* HNPP may manifest with mild, painless, distal quadriparesis. The diagnosis of HNPP may be blurred by a history of tuberculosis, tuberculostatic treatment, hepatitis, and the presence of elevated CSF-protein.

## 1. Introduction

Although hereditary neuropathy with liability to pressure palsies (HNPP) presents with a distinct phenotype on the history, clinical exam, and nerve conduction studies [1], it may be masked if diagnostic work-up suggests other causes of the clinical presentation, as in the following case.

## 2. Case Report

The patient is a 37-year-old Caucasian male from Moldavia, height 172 cm and weight 62 kg, who was referred by neurosurgeons for lumbago without radicular radiation since December 2010 and a disc prolapse at Th10/11 without indication for surgery. Urination and defecation were intact. In 1993 during the war of independence against Russia he had experienced a gunshot through the right lung, requiring resection of ribs 1 to 3 on the right side and lobectomy of the right lower lobe. Since then, he had developed occasional panic attacks, attributed to the war trauma. In 1996 he experienced a second gunshot causing a skull fracture behind the right ear and traumatic lesion of the facial, statoacoustic, and trigeminal nerves on the right side. Despite three operations, he had not been achieved complete remission and right-sided anacusis, tinnitus, peripheral facial nerve palsy, and hemihypesthesia persisted. In 1998 lung tuberculosis was detected, requiring treatment with a single drug for three years. Since then he had suffered from chronic bronchitis attributed to tuberculosis and lobectomy. He reported recurrent holocrine or occipital headache. He had no history of pressure palsies. The family history was negative for neuromuscular disorders.

Clinical neurologic examination revealed anacusis on the right side, hypoacusis on the left side, peripheral facial nerve palsy on the right side, and hemihypesthesia of the right face. There was soreness of the neck muscles and weakness M4 for finger straddling on the left and M5− on the right side, weakness for hand flexion M4+ on the left side, weakness for foot extension M5− on the left and M4+ on the right side, and weakness for foot flexion M4+ on the right side. Discrete, diffuse wasting was evident in the upper and lower limbs and tendon reflexes were generally reduced. He had left-sided hypoesthesia of digits 4 and 5 and the ulnar hand edge and right-sided hypoesthesia in a stocking-type distribution and a tendency to fall to the left back on Romberg's test.

Laboratory investigations showed positivity of antibodies against hepatitis C virus and anti-nuclear antibodies (ANA) of 1 : 1280. HCV-RNA was present in the serum with a titer of 4.5*E* + 06. ECG showed incomplete right bundle-branch-block. Lung function tests revealed basal air-trapping (compensatory overinflation of the rest of the lung). Bronchoscopy detected chronic bronchial infection but excluded active tuberculosis or silicosis. Investigation of the cerebrospinal fluid (CSF) revealed elevated protein of 76.7 mg/dL (*n*, <43 mg/dL). MRI of the thoracic spine revealed a disc prolapse at Th11/12 and MRI of the cervical spine a disc prolapse at C6/7. The results of nerve conduction studies are presented in Table 1. There were indications for demyelination such as generally prolonged distal latencies, severely reduced nerve conduction velocities, conduction blocks at sites predisposing for compression, and absence of denervation in the right anterior tibial muscle. Transcranial magnetic stimulation revealed normal latencies to the abductor halluces muscles but prolonged latencies to the abductor digiti minimi muscles with normal central conduction time, suggesting absence of myelopathy due to the thoracic or cervical disc prolapse. Search for mutations in the PMP22 gene revealed the common 1.7 mb deletion in the heterozygous form, confirming the diagnosis of HNPP.

## 3. Discussion

This case with mild quadriparesis is interesting for the multimorbidity and the unusual constellation of clinical and instrumental findings suggesting atypical CIDP, vasculitis, or side effects of tuberculostatic treatment as the main differential diagnoses of HNPP, making the correct diagnosis challenging.

The indications for HNPP in the presented patient were the nerve conduction studies, showing demyelinating neuropathy with conduction blocks at points of common pressure palsies (Table 1). Another indication for HNPP was hypoacusis. Although it was initially attributed to the trauma from the gunshot, there was no explanation for hypoacusis on the left side. Since hypoacusis is a frequent phenotypic feature of HNPP [2], the phenotype suggested hereditary neuropathy rather than any of the other possible differential diagnoses. Vasculitis of the peripheral nervous system was excluded because polyneuropathy presented without pain and was not progressive and cerebral MRI was not indicative of vasculitis either [3]. A further argument against vasculitis is the unawareness of the weakness and wasting by the patient. An argument in favour of vasculitis, however, is the presence of hepatitis B and C. Since positivity of ANA in HNPP has not been reported, elevated ANA were attributed to the hepatic infection.

CIDP was excluded, since the clinical presentation and the results of the nerve conduction studies indicated an asymmetric distribution of the neuropathy and the history was negative for previous infection and because of the long duration of the clinical manifestation, the absence of a progressive course, and negative GM1 antibodies. Elevated CSF-protein in HNPP has only rarely been reported in the literature. CSF-protein has been found elevated in a single case with HNPP [4]. Another explanation for the elevated CSF-protein could be the two prolapses at C5/6 and Th11/12 resulting in restriction of the spinal CSF circulation and thus sedimentation of the CSF-protein to the lumbar subarachnoid space.

The third differential diagnosis, complication of tuberculostatic treatment with isoniazid, was excluded, since it was given 14 years earlier and since it was not certain at all that he had received isoniazid without pyridoxine. Although polyneuropathy is a well-known side effect of such a treatment which can be prevented by concomitant pyridoxine treatment [5], it was excluded because isoniazid neuropathy is not usually associated with such low nerve conduction velocities as reported in our patient [6]. A plexus lesion was not considered as a differential diagnosis since the distribution of weakness and sensory disturbances argued against such a diagnosis. However, we have to acknowledge that other acquired neuropathies often coexist with HNPP.

The association of HNPP and a cerebral cavernoma has not been published. Thus, it remains speculative whether there was a causal link between HNPP and the cavernoma. Eosinophilia and leucopenia could be attributed to the chronic bronchitic infection or the hepatitis. Headache was interpreted as tension headache caused by the war trauma and the miserable medical and social situation. The bundle-branch block was regarded as being independent of HNPP since ECG abnormalities have not been described in these patients.

It is concluded that HNPP may manifest with mild, painless, distal quadriparesis and that the diagnosis of HNPP may be blurred by a history of gunshots, tuberculosis with tuberculostatic treatment, hepatitis, and the presence of elevated CSF-protein.

## Figures and Tables

**Table 1:** Nerve conduction studies at age of 35 y and 36 y in the presented patient.

Nerve	Age (y)	DL (ms)	CV (m/s)	Ampl (mV)	CD
Motor					
Left median (elbow–wrist)	36	5.85	54.1	13.8	No
Left median (axilla–elbow)	36	na	52.6	15.9^*∗*^	No
Right median (elbow–wrist)	36	7.12	44.2	14.1	No
Left ulnar (elbow–wrist)	36	4.34	43.9	8.2	No
Left ulnar (below/above elbow)	36	na	15.0	1.86^$^	Yes
Right ulnar (elbow–wrist)	36	4.9	52.4	10.2	No
Right ulnar (dist./prox. elbow)	36	na	19.3	9.4	No
Left ulnar (elbow–wrist)	36	4.0	32.7	6.3	No
Right ulnar (elbow–wrist)	36	4.2	36.8	6.1	No
Left peroneal (caput fibulae–ankle)	36	10.0	37.3	2.7	No
Left peroneal (dist./prox. caput fibulae)	36	na	17.5	0.24^#^	Yes
Right peroneal (caput fibulae–ankle)	36	9.92	38.8	4.0	No
Right peroneal (dist./prox. caput fibulae)	36	na	22.9	3.4	No
Left peroneal (caput fibulae–ankle)	35	11.4	37.0	2.13	No
Right peroneal (caput fibulae–ankle)	35	9.6	43.0	7.07	No
Left peroneal (dist./prox. caput fibulae)	35	na	34.0	na	No
Right peroneal (dist./prox. caput fibulae)	35	na	23.0	na	No
Left tibial (knee–ankle)	36	4.82	44.3	11.4	No
Right tibial (knee–ankle)	36	4.71	40.9	8.5	No
Right tibial (knee–ankle)	36	4.71	40.9	8.5	No
Left tibial (knee–ankle)	35	4.4	58.0	6.67	No
Right tibial (knee–ankle)	35	5.0	47.0	8.13	No
Sensory					
Left median	36	na	31.8	10.5	No
Right median	36	na	24.6	2.8	No
Right radial	36	No	28.4	7.9	No
Left sural	36	No	28.3	10.5	No
Left sural (not recordable)	35	na	na	na	na

DL: distal latency, CV: conduction velocity, Ampl: amplitude, CB: conduction block, na: not available. ^$^Amplitude difference was 79%. ^#^Amplitude difference was 91%. ^*∗*^Amplitude higher at proximal compared to distal stimulation most likely due to nonsupramaximal stimulation distally.
